# Predictors of futile recanalization in completely recanalized middle cerebral artery occlusions: multicenter study

**DOI:** 10.3389/fneur.2026.1754255

**Published:** 2026-03-09

**Authors:** Zülfikar Memiş, Erdem Gürkaş, Atilla Özcan Özdemir, Bilgehan Atılgan Acar, Muhammed Nur Ögün, Emrah Aytaç, Çetin Kürşad Akpınar, Ayşenur Önalan, Eşref Akıl, Murat Çabalar, Ayça Özkul, Ümit Görgülü, Hasan Bayındır, Zaur Mehdiyev, Şennur Delibaş Katı, Recep Baydemir, Ahmet Yabalak, Özlem Aykaç, Zehra Uysal Kocabaş, Serhan Yıldırım, Hasan Doğan, Mehmet Semih Arı, Mustafa Çetiner, Ferhat Balgetir, Fettah Eren, Alper Eren, Nazım Kızıldağ, Utku Çenikli, Aysel Büşra Şişman Bayar, Ebru Temel, Halil Alper Eryılmaz, Semanur Aksu, Emine Saygın Uysal, Hamza Gültekin, Cebrail Durmaz, Muhammet Duran Bayar, Onur Akan, Sena Boncuk Ulaş, Talip Asil

**Affiliations:** 1 Prof. Dr. Cemil Tascioglu City Hospital, Department of Neurology, Istanbul, Türkiye; 2 Department of Neurology, University of Health Sciences, Kartal Dr. Lütfi Kırdar City Hospital, Istanbul, Türkiye; 3 Department of Neurology, Faculty of Medicine, Eskisehir Osmangazi University, Eskişehir, Türkiye; 4 Department of Neurology, Faculty of Medicine, Sakarya University, Sakarya, Türkiye; 5 Department of Neurology, Faculty of Medicine, Bolu Abant Izzet Baysal University, Bolu, Türkiye; 6 Department of Neurology, Faculty of Medicine, Fırat University, Elâzığ, Türkiye; 7 Department of Neurology, Faculty of Medicine, Samsun University, Canik, Türkiye; 8 Department of Neurology, Faculty of Medicine, Dicle University, Diyarbakır, Türkiye; 9 Department of Neurology, İstanbul Başakşehir Çam Sakura City Hospital, Istanbul, Türkiye; 10 Department of Neurology, Ankara Bilkent City Hospital, Ankara, Türkiye; 11 Department of Neurology, Ankara Etlik City Hospital, Ankara, Türkiye; 12 Department of Neurology, Antalya Training and Research Hospital, Antalya, Türkiye; 13 Department of Neurology, Faculty of Medicine, Erciyes University, Kayseri, Türkiye; 14 Department of Neurology, Faculty of Medicine, Düzce University, Düzce, Türkiye; 15 Department of Neurology, Kocaeli City Hospital, Izmit, Türkiye; 16 Department of Neurology, Faculty of Medicine, Kutahya Health Sciences University, Kütahya, Türkiye; 17 Department of Neurology, Faculty of Medicine, Selçuk University, Konya, Türkiye; 18 Department of Neurology, Faculty of Medicine, Atatürk University, Erzurum, Türkiye; 19 Department of Neurology, Faculty of Medicine, Muğla Sıtkı Koçman University, Menteşe, Türkiye; 20 Deparment of Neurology, Yalova Training and Research Hospital, Yalova, Türkiye; 21 Department of Neurology, Haseki Training and Research Hospital, University of Health Sciences, Istanbul, Türkiye; 22 Department of Neurology, Sakarya Training and Research Hospital, Sakarya, Türkiye; 23 Independent Researcher (MD, Neurologist), Sakarya, Türkiye; 24 Department of Neurology, Faculty of Medicine, Biruni University, Istanbul, Türkiye

**Keywords:** acute ischemic stroke, endovascular thrombectomy, futile recanalizaition, MRS, outcome

## Abstract

**Background:**

Endovascular thrombectomy (EVT) improves outcomes and reduces mortality in acute ischemic stroke. However, despite achieving successful recanalization in most patients, a subset still experiences poor functional outcomes at 3 months. This failure, despite complete vessel reopening, is termed futile recanalization (FR). We investigated clinical and radiological predictors of FR in a multicenter cohort in Türkiye.

**Methods:**

We retrospectively analyzed 497 consecutive patients with middle cerebral artery (M1 or M2) occlusion who underwent EVT and achieved modified Thrombolysis in Cerebral Infarction (mTICI) 3 recanalization within 6 h of symptom onset at 19 stroke centers. FR was defined as a modified Rankin Scale (mRS) score ≥4 at 3 months. Clinical and radiological parameters were recorded, and logistic regression was used to identify independent predictors of FR.

**Results:**

Among 497 patients, 133 (26.7%) experienced FR despite complete recanalization. Independent predictors included older age (adjusted odds ratio [aOR] 1.07; 95% CI 1.03–1.10; *p* < 0.001), longer puncture-to-recanalization time (aOR 1.03; 95% CI 1.02–1.05; *p* < 0.001), higher admission C-reactive protein (aOR 1.01; 95% CI 1.00–1.02; *p* = 0.03), intracranial hemorrhage on 24-h CT (aOR 0.46; 95% CI 0.23–0.95; *p* = 0.04), lower collateral score (aOR 42.98; 95% CI 6.15–30.62; *p* < 0.001), and higher 24-h NIHSS score (aOR 1.34; 95% CI 1.24–1.44; *p* < 0.001).

**Conclusion:**

Even with early and complete recanalization, elderly patients and those with poor collateral circulation remain at risk for futile recanalization. Identifying these predictors can guide patient selection, procedural planning, and post-procedural management to optimize functional outcomes.

## 1 Introduction

Stroke is a global health problem and a leading cause of mortality and long-term disability worldwide (1). The advent of reperfusion therapy has revolutionized the management of acute ischemic stroke (AIS), providing significant benefits to those affected.

Endovascular thrombectomy (EVT) has been shown to improve outcomes for up to a 24-h time window in selected patients with AIS due to large vessel occlusion, providing significant benefits, including reduced long-term functional disability and mortality (2). However, despite advances such as EVT, functional outcomes remain suboptimal for a significant proportion of patients, even after achieving complete recanalization (3). This phenomenon, referred to as futile recanalization (FR), describes cases where successful vessel reopening does not translate into meaningful clinical recovery. Identifying and understanding the determinants of FR is therefore a critical unmet need in stroke research (4). Although the term FR remains a matter of debate, it is usually used to describe cases with mRS score ≥4 at 3 months, in accordance with prior literature. This threshold was deliberately chosen to exclude patients with mRS 3, who may still maintain ambulatory capacity and acceptable quality of life (5, 6).

FR is a multifactorial and complex process, and currently, the underlying mechanisms and prevention methods are still not clearly known. Investigating the risk factors associated with FR has important clinical implications, as it may improve patient selection, optimize treatment strategies, and prevent unnecessary interventions. Current relevant research results indicate that stroke severity, age, pre-stroke disability, prehospital duration, blood glucose level, comorbidities, gender, National Institutes of Health Stroke Scale (NIHSS) score, and Alberta Stroke Program Early Computed Tomography Score (ASPECTS) are associated factors for ineffective recanalization in patients who achieved satisfactory reperfusion after EVT treatment (6–9).

Nevertheless, heterogeneity in patient selection across studies has contributed to conflicting findings. While outcomes after complete recanalization [thrombolysis in cerebral infarction (TICI) 3] are consistently superior to those after incomplete recanalization (TICI 2b), it remains unclear whether this advantage is uniform across different patient subgroups (10, 11). This knowledge gap underscores the need for further investigation into which patients are most likely to benefit from successful EVT.

The present study therefore focuses specifically on patients with large vessel occlusion of the middle cerebral artery (MCA M1 or M2), aged 18–80 years, treated within 6 h of symptom onset, and achieving complete recanalization (TICI 3). By narrowing the inclusion criteria to this well-defined cohort, we aim to identify precise predictors of FR, thereby providing evidence that may guide clinical decision-making and enhance the effectiveness of EVT in real-world practice.

## 2 Materials and methods

### 2.1 Patient selection

This study is a retrospective analysis of consecutively enrolled patients with AIS who underwent endovascular treatment at 19 comprehensive stroke centers in Türkiye between January 2021 and December 2022. Prior to initiation, ethical approval was obtained from the Samsun University, with the approval number 2025/21/24. This observational study was conducted in accordance with the STROBE guidelines.

To minimize heterogeneity and focus on a well-defined population, inclusion criteria were: age 18–80 years, presentation within 6 h from last known well, pre-EVT ASPECTS ≥ 6, occlusion of the M1 or M2 segment of the middle cerebral artery, premorbid modified Rankin Scale (mRS) < 2 (12), and achievement of complete recanalization (mTICI 3) (13). The choice of thrombectomy technique (Solumbra, aspiration, or a combined approach) and the use of balloon guide catheters were left to the operator’s discretion. No patient received intravenous thrombolysis (iv-tPA) after the procedure, and no rescue stenting was performed.

Exclusion criteria included incomplete follow-up data, unsuccessful recanalization (post-mTICI < 3), occlusions other than those specified above, pre-EVT NIHSS < 6, pre-EVT ASPECTS < 6, premorbid mRS ≥ 2, and last known well exceeding 6 h.

### 2.2 Evaluation and analysis of patient data

We collected patient data including demographic characteristics, clinical data, imaging data, procedural data, and laboratory parameters. Patients’ demographic characteristics included age, sex, smoking status, alcohol consumption, hypertension, diabetes mellitus, atrial fibrillation (AF), hyperlipidemia (HL), coronary artery disease (CAD), and chronic obstructive pulmonary disease (COPD). Clinical data included admission systolic blood pressure (BP), admission diastolic BP, admission and 24th hour NIHSS (14). Imaging findings included ASPECTS (15), occluded vessels (M1, M2), collateral score, and intracranial hemorrhage. Procedural data included symptom-to-puncture time (time from onset to inguinal puncture), puncture-to-recanalization time (time from inguinal puncture to reperfusion), iv tPA, and first pass recanalization rates.

Laboratory parameters were c-reactive protein (CRP), white blood count (WBC), lymphocyte count, neutrophil count, red blood cell distribution width (RDW), platelet (PLT) count.

### 2.3 Statistics

To examine whether the continuous variables differed statistically significantly according to mRS, it was first examined whether the variables showed a normal distribution for both categories of mRS (0 = 0, 1, 2 and 3; 1 = 4, 5 and 6). The mRS = 1 group taken as FR. Since the sample size was greater than 50, the Kolmogorov–Smirnov test was used. Accordingly, the Mann–Whitney-U test was applied for continuous variables that did not show a normal distribution, and the Student’s t-test was applied for those that did show a normal distribution. Chi-square analysis was performed to examine the relationship between categorical variables and mRS. Fisher’s exact test was used in cases where the expected value in the cross table was below 5. In cases where the strength of the relationship between variables was examined, if the variables did not show a normal distribution, Spearman’s rho coefficient was used, and in cases where they did show a normal distribution, Pearson’s product–moment correlation coefficient was used.

Binary logistic regression (LR) analysis was performed to determine whether the predictor variables predicted mRS (0, 1, 2, 3 ➔0 and 4, 5, 6 ➔1) states. The data set was examined in terms of LR’s assumptions. Accordingly, whether there is a linear relationship between the continuous predictor variables and the logit transformation of the predicted (mRS) variable was examined with the Box-Tidwell approach. Tollerance value (TV) and variance inflation factor (VIF) statistics were used to examine whether there was multicollinearity between predictor variables. Accordingly, TV is in the range of 0.32–0.93 and VIF is in the range of 1.08–3.05. Accordingly, it can be said that there is no multicollinearity. At the same time, the data set was also examined for outliers. Model fit for the logistic regression analysis was assessed using the Hosmer-Lemeshow goodness-of-fit test. LR analyses were performed both single-variable and multivariable. As a result of the analysis, odds ratios, adjusted odds ratios and 95% confidence intervals, significance levels and significance levels with Bonferroni correction were examined. Analyzes were performed using SPSS 26 software and the statistical significance level was used as *p* < 0.05.

## 3 Results

Of the 497 patients, 261 (52.5%) were female and 236 (47.5%) were male, and the mean age was 65.43 ± 12.46 years. The mean ASPECTS was 8.55 ± 1.28. The symptom-to-puncture time was 194.62 ± 86.82 min. The mean NIHSS score on admission was 14.38 ± 4.75. Of the total cohort, 427 patients (85.9%) had M1 occlusion and 70 patients (14.1%) had M2 occlusion. Table 1 summarizes the basic demographic, laboratory, and clinical data of the patients. A total of 133 (26.76%) patients had FR at 3 months after acute ischemic stroke. The favorable outcome group included younger individuals compared with the FR group (*p* = 0.00). Hypertension, diabetes mellitus, and CAD were more common in the FR group (*p* value 0.03, 0.00, and 0.03, respectively). Patients in the FR group also had higher admission NIHSS scores and 24-h NIHSS scores (*p* = 0.000 and 0.000, respectively), higher CRP, WBC, and neutrophil counts (*p* = 0.000, 0.000, and 0.000, respectively), lower ASPECTS values (*p* = 0.000), longer symptom-to-puncture time (*p* = 0.000), longer puncture-to-revascularization time (*p* = 0.000), lower first-pass revascularization rates (*p* = 0.02). The FR group had lower collateral scores (*p* = 0.000) and higher 24-h hemorrhage rates (*p* = 0.000) (Table 1).

**Table 1:** Basic demographic, laboratory and clinical data of the patients.

		Total *n* = 497	MRS = 0 *n* = 364	MRS = 1(FR) *n* = 133	*p*
Patient characteristics					
Age ± sd		65.43 (12.46)	63.81 (12.95)	69.88 (9.74)	0.00**
Sex, *n*, %	Male	261 (52.5)	194 (53.3)	67 (50.4)	0.56
	Female	236(47.5)	170 (46.7)	66 (49.6)	
HT, n, %		304 (61.2)	212 (58.2)	92 (69.2)	0.03*
DM, n, %		162 (32.6)	104 (28.6)	58 (43.6)	0.00**
CAD, n, %		192 (38.6)	130 (35.7)	62 (46.6)	0.03*
COPD, n, %		47 (9.5)	32 (8.8)	15 (11.3)	0.40
AF, n, %		203 (40.8)	143 (39.3)	60 (45.1)	0.24
Alcohol, n, %		35 (7.0)	27 (7.4)	8 (6.0)	0.59
Smoke, n, %		128 (25.8)	94 (25.8)	34 (25.6)	0.95
Dyslipidemia, n, %		244 (49.1)	187 (51.4)	57 (42.9)	0.09
Laboratory findings					
CRP		16.85 (29.76)	13.99 (25.21)	24.7 (38.67)	0.00**
WBC		9.30 (4.27)	8.72 (3.05)	10.91 (6.29)	0.00**
Neutrophil		7.48 (7.85)	6.63 (4.67)	9.81 (12.81)	0.00**
Lymphocyte		2.16 (1.66)	2.1 (1.52)	2.34 (2)	0.59
PLT		230.75 (77.54)	226.79 (73.26)	241.59 (87.6)	0.06
RDW		14.88 (4.1)	14.74 (3.51)	15.26 (5.38)	0.70
Imaging findings					
ASPECTS		8.55 (1.28)	8.78 (1.18)	7.94 (1.35)	0.00**
Vessel occlusion	M1, n, %	427 (85.9)	312 (85.7)	115 (86.5)	0.830
	M2, n, %	70 (14.1)	52 (14.3)	18 (13.5)	
Colleteral score	0	50 (10.1)	18 (4.9)	32 (24.1)	0.00**
	1	158 (31.8)	84 (23.1)	74 (55.6)	
	2	221 (44.5)	196 (53.8)	25 (18.8)	
	3	68 (13.7)	66 (18.1)	2 (1.5)	
ICH, n, %		112 (22.5)	58 (15.9)	54 (40.6)	0.00**
Clinical findings					
NIHSS at admission		14.38 (4.75)	13.51 (4.46)	16.77 (4.74)	0.00**
24^th^ Hour NIHSS		8.62 (6.66)	6.21 (5.16)	15.22 (5.79)	0.00**
Procedural data					
Symptom-to-puncture (min)		194.62 (86.82)	186.70 (85.4)	216.29 (87.3)	0.00**
Puncture-to-recanalization (min)		41.78 (22.00)	39.72 (20.2)	47.4 (25.56)	0.00**
IV tPA, n, % ***		152 (30.6)	114 (31.3)	38 (28.6)	0.56
First pass recanalization, *n*, %		274 (55.1)	212 (58.2)	62 (46.6)	0.02*

sd, standard deviation; mRS, modified Rankin Scale; FR, futile recanalization; HT, Hypertension; DM, diabetes mellitus; CAD, coronary artery disease; COPD, chronic obstructive pulmonary disease; AF, atrial fibrilation; CRP, C-reative protein; WBC, white blood cell; PLT, platelet; RDW, red blood cell distribution width; ICH, intracerebral hemorrhage; NIHSS, National Institute of Health Stroke Scale; min, minute; IV, intravenous; tPA, tissue plasminogen activator.

*p < 0.05, **p < 0.01, ***IV-tPA refers to bridging therapy administered prior to endovascular treatment; no iv-tPA was given after EVT.The bold values represent statistically significant values.

Multivariate logistic regression analysis identified several independent predictors of futile recanalization. Older age (aOR 1.07, 95% CI 1.03–1.10, *p* = 0.00), elevated C-reactive protein (CRP) (aOR 1.01, 95% CI 1.00–1.02, *p* = 0.03), and the presence of symptomatic intracerebral hemorrhage at 24 h (aOR 0.46, 95% CI 0.23–0.95, *p* = 0.04) were significantly associated with FR (Table 2). However, after applying the Bonferroni correction for multiple comparisons, older age, poor collateral scores, 24-h NIHSS, and puncture-to-recanalization time remained robust independent predictors (adjusted *p* < 0.001). Admission CRP levels and 24-h ICH findings did not meet the stringent significance threshold after correction (adjusted *p* = 0.60 and *p* = 0.80, respectively) and should be interpreted as exploratory findings. In addition, patients with poor collateral circulation (score 0–1) had markedly higher odds of FR (aOR 61.32, 95% CI 7.69–489.12, *p* = 0.00 for score 0; aOR 42.98, 95% CI 6.15–300.62, *p* = 0.00 for score 1). Furthermore, higher NIHSS scores at 24 h (aOR 1.34, 95% CI 1.24–1.44, *p* = 0.00) and longer puncture-to-recanalization time (aOR 1.03, 95% CI 1.02–1.05, *p* = 0.00) were independent predictors of FR (Table 2). The multivariable model demonstrated good calibration and fit to the data (Hosmer-Lemeshow test: $\chi^{2}=$ 7.60, df = 8, *p* = 0.47).

**Table 2:** Futile recanalization related parameters according to the logistic regression analysis.

	aOR(95%CI)	*p*	Adjusted *p* (Bonferroni)
Age	1.07 (1.03–1.1)	0.00**	0.00**
CRP	1.01 (1–1.02)	0.03*	0.60
ASPECTS	0.85 (0.65–1.1)	0.21	1.00
ICH at 24^th^ hour CT	0.46 (0.23–0.95)	0.04*	0.80
Collateral Score 0	61.32 (7.69–489.12)	0.00**	0.00**
Collateral Score 1	42.98 (6.15–300.62)	0.00**	0.00**
NIHSS at admission	0.96 (0.88–1.05)	0.36	1.00
24^th^ Hour NIHSS	1.34 (1.24–1.44)	0.00**	0.00**
Symptom-to-puncture time (min)	1 (1–1)	0.90	1.00
Puncture-to-recanalization time (min)	1.03 (1.02–1.05)	0.00**	0.00**
EVT technique [Adapt]	1.21 (0.53–2.74)	0.65	1.00
EVT technique [Solumbra]	1.54 (0.67–3.55)	0.31	1.00
First pass recanalization	0.92 (0.27–3.08)	0.89	1.00

aOR, adjusted odds ratio; HT, hypertension; CAD, coronary artery disease; CRP, C-reative protein; WBC, white blood Cell; ASPECTS, Alberta Stroke Program Early Computed Tomography Score; ICH, intracranial hemorrhage; CT, computed tomography; NIHSS, National Institute of Health Stroke Scale; min, minute; EVT, endovascular thrombectomy.

*p < 0.05, **p < 0.01, Hosmer-Lemeshow goodness-of-fit test: $\chi^{2}$ =7.60, df = 8, *p* = 0.47.The bold values represent statistically significant values.

## 4 Discussion

AIS poses a significant economic and social burden to society due to its high morbidity and disability. The key to treating AIS is restoring blood flow and halting neuronal damage as quickly as possible. EVT has been shown to be the most successful treatment in patients with anterior circulation AIS due to LVO (2).

Recent studies have moved away from using the term “futile recanalization” because some of these patients may still have a certain quality of life and therefore endovascular intervention is not considered futile (5). A patient with a mRS of 3 may be dependent on others for daily activities but remain independent for walking. This is considered a significant result for patients (5, 6). In the literature, definitions of poor functional outcome vary, with some studies considering mRS ≥ 3 at 3 months as poor outcome, while others use mRS ≥ 4 (16–18). In the present study, FR was defined as an mRS score of 4–6 at 3 months. Based on this definition, the rate of FR in our cohort was 26.7% (*n* = 133). This proportion is lower than that reported in many previous studies (19–21). For example, one study published in 2024 reported a FR rate of 66.6% (19), while another reported 40% (20). A likely explanation for the lower rate observed in our study is the stricter definition of FR (mRS 4–6), as well as the inclusion of only patients who achieved complete reperfusion (TICI 3) within 6 h of symptom onset, in contrast to broader inclusion criteria used in other studies.

To the best of our knowledge, this is the first study to exclusively investigate patients with middle cerebral artery occlusion who achieved TICI 3 reperfusion within 6 h of symptom onset. In this cohort, older age, elevated admission CRP levels, higher 24-h NIHSS, presence of ICH, prolonged puncture-to-recanalization time, and poor collateral scores were identified as independent predictors of FR. Previous studies have shown that, although EVT in elderly patients achieves recanalization rates comparable to those in younger patients, clinical outcomes at 3 months are generally worse in older populations (22, 23). This disparity may be explained by the higher burden of comorbidities, impaired collateral circulation, and greater prevalence of vascular pathology in the elderly. The rationale for the 80-year age limit in our cohort was to ensure a relatively homogeneous study population and to align with the inclusion criteria of pivotal randomized controlled trials of endovascular thrombectomy. Moreover, advanced age is associated with a higher burden of comorbidities, greater baseline brain atrophy, and impaired collateral circulation, all of which may independently affect functional outcomes. Furthermore, increasing baseline brain atrophy with age has been associated with a higher risk of FR (24). Cerebral atrophy reflects a reduced neuronal reserve and may also indicate a compromised microvascular network that is less resilient to the hemodynamic changes following the mechanical thrombectomy. This diminished vascular and neuronal reserve can exacerbate ischemic injury, limit functional recovery, and contribute to the potential leading to a higher incidence of FR. Mistry et al. (25) demonstrated that severe white matter disease predicts poorer outcomes after endovascular treatment. Nevertheless, current evidence suggests that imposing a strict upper age limit for EVT is not justified.

Consistent with our findings, recent studies have demonstrated that elevated pre-procedural CRP is an independent predictor of FR (25, 26). The association between CRP and stroke outcomes may be explained by its role in activating thrombo-inflammatory pathways, initiating local inflammatory responses, and subsequently triggering complement activation, which exacerbates tissue damage and worsens outcomes (27).

In our cohort, admission NIHSS was not associated with FR; however, a strong independent association was observed between elevated 24-h NIHSS and FR. This observation aligns with the majority of recent studies, which emphasize that 24-h NIHSS provides robust prognostic value for predicting long-term outcomes and FR (28, 29). In contrast, other studies have reported that admission NIHSS also influences FR (19, 30).

Post-recanalization ICH has been associated with poor clinical outcome and mortality in many recent studies (31, 32). Our results corroborate these findings, identifying ICH as an independent predictor of FR.

A longer puncture-to-recanalization time was confirmed to be associated with FR by Saver et al. (33, 34). Consistent with previous studies, we suggest that a delayed puncture-to-recanalization time is significantly associated with FR. Although the operator’s experience and skill are very important in reducing puncture-to-recanalization time, factors such as clot burden and anatomical difficulties may prolong the time and increase the number of endovascular interventions.

The prognostic importance of collateral circulation has been consistently emphasized across multiple trials. In a post-hoc analysis of the SWIFT study, one of the earliest EVT trials, better collateral status was associated with improved reperfusion and favorable 3-month outcomes (35). Similarly, the IMS III study of 331 patients demonstrated that good collateral flow correlated with successful recanalization, reperfusion, and improved outcomes (36). Findings from the TREVO2 trial confirmed collateral status as a predictor of favorable outcomes (37), while the MR RESCUE study highlighted its role in determining clinical prognosis regardless of treatment modality (38). Furthermore, a post-hoc collateral analysis of the DAWN trial identified favorable collateral scores as independent predictors of good functional outcome (39). Our findings are consistent with this growing body of evidence, further supporting collateral status as a significant independent predictor of FR.

In our study, poor collateral status (Score 0 and 1) was found to be the strongest independent predictor of futile recanalization, with notably high adjusted odds ratios. While the magnitude of these effect sizes and the accompanying wide confidence intervals may suggest a degree of sparse data bias due to the distribution of cases across sub-categories, the direction and strength of this association are biologically plausible. This finding aligns with the ‘collateral-centric’ understanding of stroke, where the absence of robust collateral flow accelerates irreversible tissue damage despite successful macrovascular recanalization.

In addition to the variables that were concluded to be independent predictors of FR in our study, there are also variables that are associated with FR in the literature but not in our study. HT (31), DM (31), Neutrophil-Lymphocyte ratio (NLR) (40), AF (31), admission NIHSS (19), ASPECT Score (31) are some of these. This discrepancy may suggest that in patients with early and complete recanalization, fewer variables exert an impact on the occurrence of FR.

This study has several limitations. First, although data were collected from 19 comprehensive stroke centers, assessments of TICI score and collateral flow were performed by local operators at each center. This introduces potential inter-operator variability, as discrepancies are known to exist between operator-based evaluations and those conducted by independent core laboratories, with a tendency among operators to overestimate reperfusion (41). Second, although EVT can be performed up to 24 h after symptom onset, only patients treated within the early time window were included. While this selection ensured a more homogeneous cohort, it may limit the generalizability of the findings, as different results might be observed in patients treated between 6 and 24 h. Additionally, the inclusion of only patients with MCA occlusion, while providing a uniform study population, restricts the applicability of the results to other vascular territories. Finally, the notably high adjusted odds ratios and wide confidence intervals observed for collateral scores in our multivariable model should be interpreted with caution. This finding likely reflects a degree of sparse data bias due to the specific distribution of cases within these sub-categories, representing a statistical limitation of the study despite the biological plausibility of the results.

## 5 Conclusion

Futile recanalization remains a significant challenge in patients undergoing EVT for acute ischemic stroke, even among those who achieve complete reperfusion within the early time window. Our study identifies older age, elevated CRP levels, higher 24-h NIHSS, post-procedural ICH, prolonged puncture-to-recanalization time, and poor collateral circulation as independent predictors of FR. Recognizing these factors may aid clinicians in optimizing patient selection, procedural planning, and post-procedural management, ultimately improving functional outcomes. Further studies are warranted to validate these predictors and explore strategies to mitigate the risk of FR.

## Data Availability

The datasets analyzed during the study are not publicly available due to regional ethical reasons. Requests to access the datasets should be directed to ZM, zulfikarmemis1@gmail.com.
